# Connectivity Features for Identifying Cognitive Impairment in Presymptomatic Carotid Stenosis

**DOI:** 10.1371/journal.pone.0085441

**Published:** 2014-01-15

**Authors:** Chun-Jen Lin, Pei-Chi Tu, Chang-Ming Chern, Fu-Jung Hsiao, Feng-Chi Chang, Hsien-Lin Cheng, Chih-Wei Tang, Yi-Chung Lee, Wei-Ta Chen, I-Hui Lee

**Affiliations:** 1 Department of Internal Medicine, Taipei Veterans General Hospital Hsinchu branch, Hsinchu, Taiwan; 2 Institute of Brain Science, National Yang-Ming University, Taipei, Taiwan; 3 Department of Medical Education & Research, Taipei Veterans General Hospital, Taipei, Taiwan; 4 Department of Neurology, Taipei Veterans General Hospital, Taipei, Taiwan; 5 Department of Education and Research, Taipei City Hospital, Taipei, Taiwan; 6 Departement of Radiology, Taipei Veterans General Hospital, Taipei, Taiwan; 7 Department of Physical Medicine and Rehabilitation, Taipei Medical University Hospital, Taipei, Taiwan; 8 Department of Internal Medicine, Far Eastern Memorial Hospital, New Taipei, Taiwan; 9 School of Medicine, National Yang-Ming University, Taipei, Taiwan; Tianjin Medical University General Hospital, China

## Abstract

Severe asymptomatic stenosis of the internal carotid artery (ICA) leads to increased incidence of mild cognitive impairment (MCI) likely through silent embolic infarcts and/or chronic hypoperfusion, but the brain dysfunction is poorly understood and difficult to diagnose. Thirty cognitively intact subjects with asymptomatic, severe (≧70%), unilateral stenosis of the ICA were compared with 30 healthy controls, matched for age, sex, cardiovascular risk factors and education level, on a battery of neuropsychiatric tests, voxel-based morphometry of magnetic resonance imaging (MRI), diffusion tensor imaging and brain-wise, seed-based analysis of resting-state functional MRI. Multivariate regression models and multivariate pattern classification (support vector machines) were computed to assess the relationship between connectivity measures and neurocognitive performance. The patients had worse dizziness scores and poorer verbal memory, executive function and complex visuo-spatial performance than controls. Twelve out of the 30 patients (40%) were considered to have MCI. Nonetheless, the leukoaraiosis Sheltens scores, hippocampal and brain volumes were not different between groups. Their whole-brain mean fractional anisotropy (FA) was significantly reduced and regional functional connectivity (Fc) was significantly impaired in the dorsal attention network (DAN), frontoparietal network, sensorimotor network and default mode network. In particular, the Fc strength at the insula of the DAN and the mean FA were linearly related with attention performance and dizziness severity, respectively. The multivariate pattern classification gave over 90% predictive accuracy of individuals with MCI or severe dizziness. Cognitive decline in stroke-free individuals with severe carotid stenosis may arise from nonselective widespread disconnections of long-range, predominantly interhemispheric non-hippocampal pathways. Connectivity measures may serve as both predictors for cases at risk and therapeutic targets for mitigating vascular cognitive impairment.

## Introduction

Patients with “asymptomatic” severe carotid stenosis, conventionally defined by free of stroke or transient ischemic attack[1], have been found to have increased susceptibility to cognitive impairment in memory and complex visuospatial perception[1]–[4]. Accumulating data suggest that silent embolic infarctions[5] and perfusion insufficiency[6] may both contribute to such mild cognitive impairment (MCI), [7] namely a subtype of vascular cognitive impairment no dementia (VCIND). However, the consequent neural dysfunction has yet to be elucidated in human studies. In an experimental murine model of unilateral carotid artery occlusion, the animals had impaired object recognition that was associated with microstructural demyelination and axonopathy of the corpus callosum and frontal-subcortical circuits[8]. We therefore hypothesize that severe “asymptomatic” carotid stenosis may impose risks on subclinical neurocognitive dysfunction and network disconnections. Resting-state functional magnetic resonance imaging (rs-fMRI) and diffusion tensor imaging (DTI) have been increasingly used to assess functional and microstructural connectivity in neuropsychiatric disorders[9], [10]. While the blood oxygenation level-dependent (BOLD) signals of rs-fMRI may be affected by impaired neurovascular coupling and cerebrovascular reactivity in cerebrovascular diseases[11], [12], BOLD-independent DTI can complementarily differentiate a loss in structural connectivity from a loss of cerebral blood flow. We identified for the first time that patients with severe unilateral asymptomatic carotid stenosis (n = 17) relative to healthy controls (n = 26) had mild cognitive impairments accompanied by more ipsilateral lacunes and functional disconnections predominantly between inter-hemispheric homologous regions of interest (ROI) selected a priori in the frontoparietal network (FPN) and, to a less degree, in the ipsilateral default mode network (DMN)[13]. These results suggest that unilateral severe carotid stenosis not only affects ipsilateral focal neural circuits but also disrupts remote interhemispheric connections. However, brain-wise connectivity measures beyond our prespecified homologous ROI pairs in three selected networks have not been explored. Additionally, we noted measurable connectivity increments at three months following successful carotid revascularization (defined by residual stenosis <50% without peri-procedural complications) in an uncontrolled pre-post comparison (n = 10)[13], which were accompanied by an insignificant trend of cognitive improvements as others have similarly reported[14], [15]. Interventional revascularization for “asymptomatic” carotid stenosis has been an unsettled issue. Large prospective controlled studies are warranted to weigh the risks and benefits not only for stroke prevention but also for mitigating VCIND in these patients.

A lack of commonly agreed tools and standards has prevented patients with MCI/VCIND from receiving early treatment. Conventional MRI and white matter hyperintensities (leukoaraiosis) are unsatisfactory for detecting abnormalities correlated with functional changes in these patients. Advanced connectivity MRI measures may provide insights into how to best identify which patients derive significant benefits from revascularization for cognitive and network abnormalities at earlier stages of the illness. Here, we sought a comprehensive assessment of the most significant features of brain-wise connectivity (rs-fMRI and DTI) and volumetric measures (voxel-based morphometry, VBM) in patients with severe unilateral asymptomatic carotid stenosis. Moreover, we employed multivariate regression analyses and support vector machines (SVM) classification to determine whether connectivity markers may be used to aid early diagnosis of MCI/VCIND and identify the most appropriate patients for revascularization at presymptomatic stages.

## Materials and Methods

### Subjects and neuropsychological tests

We consecutively enrolled cognitively intact and neurologically normal subjects with asymptomatic, unilateral stenosis of the extracranial internal carotid artery (ICA) incidentally discovered by ultrasound examinations at the Neurology outpatient clinic of Taipei Veterans General Hospital between March 2010 and January 2013. The inclusion criteria were age between 55 and 80 years and ICA stenotic degree ≧70% by both duplex ultrasonography[16] and MR angiography (according to North American Symptomatic Carotid Endarterectomy trial criteria)[17] of the neck and head. The exclusion criteria included the presence of contralateral ICA stenosis ≧50%, posterior circulation diseases (subclavian steal and vertebrobasilar insufficiency), dementia (mini-mental status examination score (MMSE) <27, see Results for a further discussion), functional disability (modified Rankin Scale ≧3) and other major neuropsychiatric diseases (such as major depression, Parkinson's disease, multiple sclerosis, encephalitis and severe head injury with consciousness change) or severe systemic diseases (such as congestive heart failure, chronic obstructive pulmonary disease, cirrhosis, renal failure and malignancy with distant metastasis). Healthy controls without carotid stenosis as determined by ultrasound examinations were also enrolled from the same clinic or from the Taipei community through advertisements. The medications of both groups were recorded (antiplatelets, anticoagulants, hypnotics, anticholinergics et al.). Written informed consent was obtained from each participant before the experiment.

All participants were assessed by a blinded examiner on a battery of neuropsychological tests, including the dizziness handicap inventory (DHI, a most commonly used standardized questionnaire originally developed to assess the self-perceived handicapping effects from vestibular dysfunction)[18], MMSE, Taiwan Geriatric Depression Scale (TGDS)[19], memory tests (digit span and auditory verbal learning tests including immediate and 15 minute-delayed recall of 12 items, i.e., the verbal selective reminding test)[20], executive function tests (the modified trail making test A and B and the Stroop color-word test), an attention test (the symbol digit test), complex visuospatial perception tests (the modified complex figure test with copy and recall). This study was approved by the ethics committee of the Taipei Veterans General Hospital (VGHIRB No. 98-08-04A, No. 2011-12-009GA, No. 2012-01-016AC).

### MRI acquisition

We used a 3.0 GE Discovery 750 MRI scanner with foam padding and earplugs to restrict head motion and reduce scanner noise. All subjects were instructed to hold still, keep their eyes open and think of nothing. All of the images were acquired along the anterior–posterior commissural plane, as identified by multiplanar T1-weighted BRAVO anatomical images (repetition time, TR = 12.2 ms; echo time, TE = 5.2 ms; flip angle = 12°; voxel size = 1×1×1 mm; field of view, FoV = 256×256 mm). A fluid attenuation inversion recovery (FLAIR) sequence was also acquired for rating white matter lesions (leukoaraiosis). For DTI, we used a single-shot diffusion spin-echo echo-planar imaging sequence (TR/TE = 9500/85.6 ms; thickness = 2 mm; matrix = 128×128; FoV = 256×256 mm; 30 directions). For fMRI, we recorded BOLD signals from a task-free run (124 time points/372 s) of a gradient-echo echo-planar imaging sequence (TR/TE = 3000/30 ms; flip angle = 90°; FoV = 222×222 mm; thickness = 3 mm).

### MRI analysis

A blinded neurologist and a neuroradiologist with specialty and subspecialty certificates evaluated all of the images. The semiquantitative Sheltens rating scale was used to evaluate the degree of leukoaraiosis[21]. For imaging analysis, the hemisphere ipsilateral to ICA stenosis was flipped to the left side along the mid-sagittal plane. We analyzed T1-weighted anatomical images to calculate hippocampal volume (by manually outlining bilateral hippocampi and averaging the volumes for each subject)[22], [23] and brain volume using the VBM approach[24]. Statistical Parametric Mapping software (SPM8, Wellcome Department of Cognitive Neurology in London) was utilized. Gray and white matters were segmented with default settings and spatially normalized to a group specific template in Montreal Neurological Institute (MNI) space using a diffeomorphic image registration toolkit (DARTEL) in 1.5 mm cubic resolution. After concentration variations were smoothed by convolution with an isotropic Gaussian kernel (half width: 5 mm), the gray and white matter volumes were compared between groups by two-sample t-tests with a threshold of *P*<0.05. For the DTI analysis, voxel-wise FA was analyzed following preprocessing with tract-based spatial statistics from the FMRIB Software Library (FSL 3.2, http://www.fmrib.ox.ac.uk/fsl) as previously described[13]. The FA values between groups were compared by two-sample t-tests using a threshold of *P*<0.05 with family-wise error rate correction for multiple comparisons (n = 5,000 random permutations).

For the fMRI analysis, a preprocessing procedure was performed as previously described[25]. ROIs within a 4 mm radius were predefined ipsilateral to ICA stenosis to represent the seed regions for six resting-state networks[25]–[27]. The MNI coordinates of the six seeds were as follows: left frontal eye field (FEF, −26, 6, 48) in the dorsal attention network (DAN), left middle frontal gyrus (MFG, −45, 29, 32) in the FPN, left primary motor cortex (M1, −41, −20, 62) in the sensorimotor network (SMN), posterior cingulate cortex (PCC, 0, −50, 22) in the DMN, dorsal anterior cingulate cortex (dACC, −1, 10, 46) in the salience network (SN) and left primary visual cortex (V1, −4, −81, −10) in the visual network (VN). Pearson correlation coefficients (r) for temporal correlations between the BOLD signals from each ROI and brain-wise voxels were calculated using a Fisher's r to z transformation. Voxel-wise z values, i.e., functional connectivity (Fc), from a single ROI per network were computed with one-sample t tests by using SPM8. For within-group analysis and 3D visualization of the networks, we applied Computerized Anatomical Reconstruction and Editing Toolkit (Van Essen Laboratory, Department of Anatomy and Neurobiology, Washington University School of Medicine, St. Louis, Missouri, USA). For between-group analysis, z maps of individual networks were compared by two-sample t-tests, and significance was defined as false discovery rate-adjusted, *Q*<0.05 by combining voxel intensity (T values) and the cluster extent above a threshold size of 50 voxels.

### Statistical analysis of demographic/neuropsychological variables, multivariate regression models and support vector machines

We used SPSS software (version 18.0, Chicago, USA) for all of the statistical analyses. Categorical variables were compared using Chi-squared or Fisher exact tests if the expected number was ≦5. The neuropsychological and leukoaraiosis scores were compared by two-sample t-tests between groups. Significance was defined as *P*<0.05, corrected by the Bonferroni method for 11 cognitive measures.

To evaluate relationships between connectivity measures and neuropsychological presentations, we performed multivariate regression analyses in all of the subjects. The dependent variables were respective neuropsychological measures (including 11 cognitive items and the DHI score) and the independent variables were age, sex, education levels, presence or absence of ICA stenosis (group), mean FA and the z values of the peak voxels representing the most significantly affected clusters of each resting networks, if any. For simplified graph demonstration, single variate linear regression between significantly correlated connectivity measures and neuropsychological scores was shown. Besides, to evaluate the relationship between FA and Fc, correlation tests were analyzed between mean FA and the significantly affected z value from each network in all subjects.

To identify individuals with MCI/VCIND and severe dizziness from all subjects, we employed a multivariate pattern classification by using libSVM v.3.0 (www.csie.ntu.edu.tw/~cjlin/libsvm/)[28]. Briefly, a radial basis function kernel matrix was generated. The classification features included age, sex, education levels, presence or absence of ICA stenosis (group), mean FA and the z values of the peak voxels within the most significantly affected clusters of respective resting networks. A cut-off value of delayed verbal recall or DHI scores as 1.5 SD below the mean of healthy control group was used to classify individuals with MCI/VCIND or severe dizziness. Because of the small sample size, we used a leave-one-out cross-validation strategy to evaluate the classification/predictive accuracy (number of correct classifications/number of all attempted classifications), i.e. data from all but one subject were used for model training and data from the remaining one was then used to test the classifier. This procedure was repeated until each subject was used for testing once. Thereafter, we estimated the diagnostic power of receiver operating characteristic curve, the percentage of area under the curve (AUC). To identify the most discriminative features of our classifiers, we calculated a weighting scheme[29] for each feature and removed those with the least weighting one by one. The minimum feature selection required for the best classification/predictive accuracy (at least over 90%) was adopted.

## Results

Thirty-eight patients and 30 healthy controls were consecutively enrolled from the same clinic, but eight patients were excluded because of the presence of contralateral carotid stenosis (n = 4) or possible mild dementia (MMSE<27; n = 4). Therefore, 30 patients participated the study. Among the participants, 15 patients and 26 healthy controls had been reported in our previous study on homologous ROI selected a priori in three resting networks[13]. The demographic characteristics, including age, sex, average education, depression scores, risk factor prevalence, leukoaraiosis scores (Table 1) and the medication use (data not shown) were insignificantly different between groups. The patients had lower global MMSE scores than the healthy controls (mean ± standard deviation (SD): 28.23±1.11 vs. 29.29±0.69, *P* = 0.01). Nevertheless, their scores were still within the normal aging range suggested by a previous study of 326 normal Taiwanese individuals (MMSE: 28.5±1.3; average age (73.1) and average education (11 years) were similar to our participants)[30]. In addition, the patients had significantly worse dizziness scores and poorer cognitive performance on verbal memory, executive tasks and complex visuospatial perception tests. However, attention was not different between the groups (Table 1). A cut-off value of MCI/VCIND has yet to be defined, but a single cognitive test performance of 1.5 SD below the control mean has been accepted and used. In large cohorts, this means that such deficits occur in less than 7% of the healthy population. We used a delayed verbal recall test (patients vs. controls: 8.00±2.78 vs. 10.84±1.39, *P*<0.01) and considered a cut-off value of ≦8 (1.5 SD below the mean of the healthy control group) as relevantly impaired. This reference value was comparable to the reported values using the similar test in 267 normal European adults (age range 18–91 years)[31]. Thus, in large cohorts, this means that such deficits occur in less than 7% of the healthy population. Twelve out of 30 patients (40%) and none of the controls met this MCI/VCIND criteria, although the actual proportion may be slightly lower considering adjustment for age[31].

**Table 1:** Basic demographic, neuropsychological and structural MRI characteristics of healthy controls and patients with asymptomatic carotid stenosis.

	Patients (n = 30)	Controls (n = 30)	*P*
Age (years)	70.80±8.26	69.81±5.79	0.590
Male: Female (male%)	17:13 (56.7)	16:14 (53.3)	0.510
Education (years)	10.20±4.97	10.74±3.76	0.632
Depression score (TGDS)	5.67±2.32	4.65±3.23	0.161
Risk factors (%)			
Hypertension	53.3	43.3	0.093
Diabetes mellitus	33.3	16.7	0.075
Hypercholesterolemia	33.3	25.8	0.150
Atrial fibrillation	6.7	3.3	0.399
Smoking	23.3	16.7	0.491
Stenotic side and degree (%)			
Left (n = 15)	79.23±9.96	N/A	
Right (n = 15)	83.82±11.25	N/A	
Dizziness Handicap Inventory	22.73± 16.79	6.52±12.02	<0.01*
Mini Mental Status Examination	28.23±1.10	29.29±0.69	0.01†
Verbal memory tests			
Forward digit span	7.38±1.63	8.06±1.09	0.76
Backward digit span	3.97±1.50	5.29±1.16	0.01†
Immediate recall	44.00±11.82	54.23±7.42	<0.01†
Delayed recall	8.00±2.78	10.84±1.39	<0.01†
Attention tests			
Symbol digit test	43.37±23.92	57.19±12.54	0.10
Executive function tests			
Modified trail making test A	20.39±13.32	11.19±4.53	0.01†
Modified trail making test B	52.55±33.09	32.23±11.79	0.07
Stroop test	31.28±14.59	40.68±11.16	0.07
Complex visuospatial perception			
Modified complex figure test (copy)	15.46±1.75	16.71±0.90	0.02†
Modified complex figure test (recall)	9.25±4.18	12.32±3.03	0.02†
Sheltens leukoaraiosis score	6.20±3.23	5.71±3.15	0.596
Hippocampal volume (ml)	3.10±0.57	3.18±0.42	0.831
Normalized gray matter volume (ml)	717.38±55.88	716.55±65.15	0.955
Normalized white matter volume (ml)	473.35±33.76	475.01±45.47	0.876
Mean FA	0.49±0.03	0.56±0.02	0.001*

Values expressed as the mean ± standard deviation. *P<0.05 is considered significant. †Bonferroni corrected *P*<0.05 for neuropsychiatric tests. N/A: not applicable; TGDS: Taiwan Geriatric Depression Scale.

**Figure 1:**
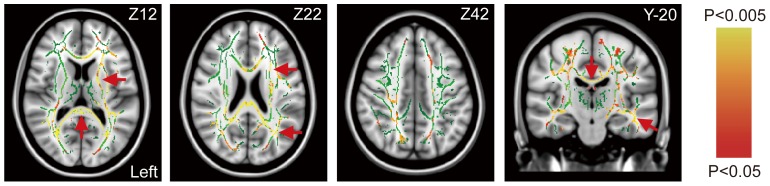
Horizontal (left three) and coronal (right) fractional anisotropy (FA) maps. The carotid stenotic side is set to the left in all patients. The white matter skeleton derived from the controls is shown in green. Note the significant decrements of FA (red-yellow in the color bar) in the patients, particularly at the splenium, the lateral basal ganglion and frontoparietal regions ipsilateral to the stenosis (red arrows). There was no notable increment of FA compared to controls.

There were no differences in leukoaraiosis scores (*P* = 0.596), hippocampal volume (*P* = 0.831), whole-brain gray matter (*P* = 0.955) or white matter volume (*P* = 0.876) between groups. However, the microstructural white matter integrity, e.g., the mean FA value, of the patients was significantly impaired relative to controls (0.49±0.03 vs. 0.56±0.02, *P* = 0.001) (Table 1). Distributed and asymmetric FA decrements, particularly in the ipsilateral lateral basal ganglion, frontoparietal regions and splenium, were noted in the voxel-wise between-group FA comparisons (Figure 1).

**Figure 2:**
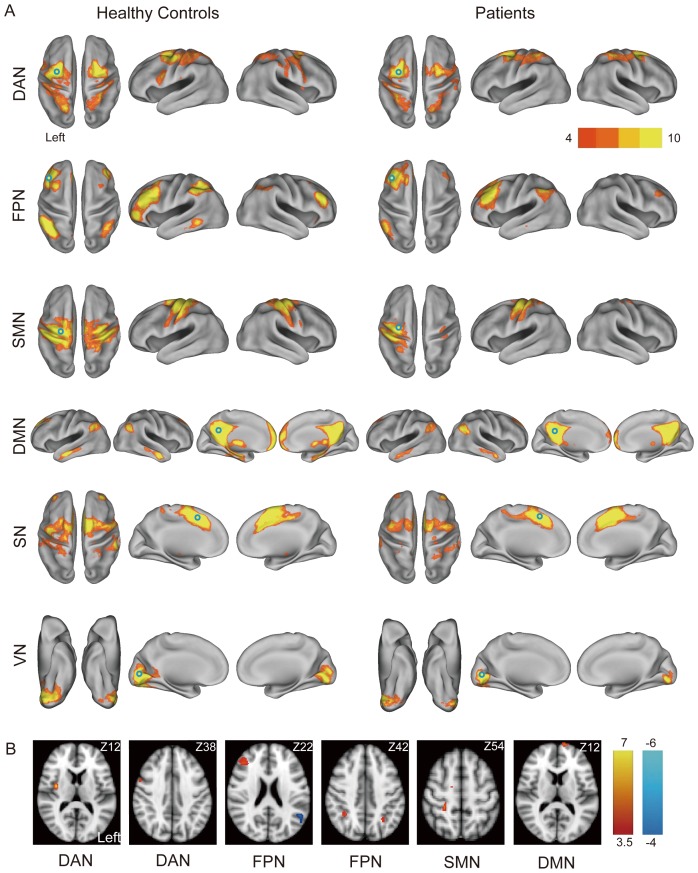
Comparisons of six resting-state functional networks between healthy controls and patients. (A) A within-group analysis of resting-state networks in healthy controls and patients. Hollow circles indicate the predefined ROIs for individual networks. (B) Group comparisons of respective resting-state networks. Clusters with significant decrements of functional connectivity in the patients are shown in red-yellow; increments of functional connectivity are shown in blue. The carotid stenotic side was flipped to the left. Color bars represent T values. DAN: dorsal attention network; FPN: frontoparietal network; SMN: sensorimotor network; DMN: default mode network; SN: salience network; VN: visual network.

From the perspective of functional networks, the patients had disrupted and more asymmetric networks of the DAN, FPN, SMN and DMN than the controls (Figure 2A), which suggests that long-range interhemispheric connections were profoundly affected. The SN and VN were relatively preserved in the patients. Among these disrupted foci (clusters), the contralateral (in relation to ICA stenosis) insula in the DAN and the contralateral MFG in the FPN were the top two affected regions, followed by the contralateral dorsolateral prefrontal cortex (DLPFC) in the DAN, the bilateral inferior parietal lobules (IPL) in the FPN, the contralateral primary somatosensory cortex (S1), the contralateral supplementary motor cortex (SMC) in the SMN and the ipsilateral medial prefrontal cortex (MPF) in the DMN (Figure 2B; also see Table 2). Moreover, the ipsilateral superior temporal lobule (STL) in the FPN showed increased connectivity, which may reflect less anti-correlated activity within the network or compensatory activity in response to a nearby network disruption (Figure 2B). The latter is less likely because the patients did not have a notable FA increase on DTI.

**Table 2:** Brain-wise differences in functional connectivity of brain networks between patients and controls.

Networks	Coordinates (MNI)			*Q*	Cluster size	T score
	x	y	z			
**Dorsal Attention Network**						
Right insula	36	−4	12	<0.001	573	6.65
Right dorsolateral prefrontal cortex	54	6	38	0.001	207	4.67
**Frontoparietal Network**						
Right middle frontal gyrus	44	34	22	<0.001	307	4.58
Right inferior parietal lobule	36	−48	44	0.001	228	4.49
Left inferior frontal gyrus	−52	42	4	0.006	140	5.12
Left inferior parietal lobule	−28	−56	40	0.016	106	4.49
Left supramarginal gyrus	−52	−54	22	<0.001	237	−4.97
**Sensorimotor Network**						
Right primary sensory cortex	24	−32	54	0.001	245	4.47
Right supplementary motor cortex	10	−26	60	0.031	116	4.39
**Default Mode Network**						
Left medial prefrontal cortex	−12	70	12	0.003	193	4.36

The carotid stenosis is flipped to the left side in all patients. A false discovery rate-corrected *Q*<0.05 is considered significant.

**Figure 3:**
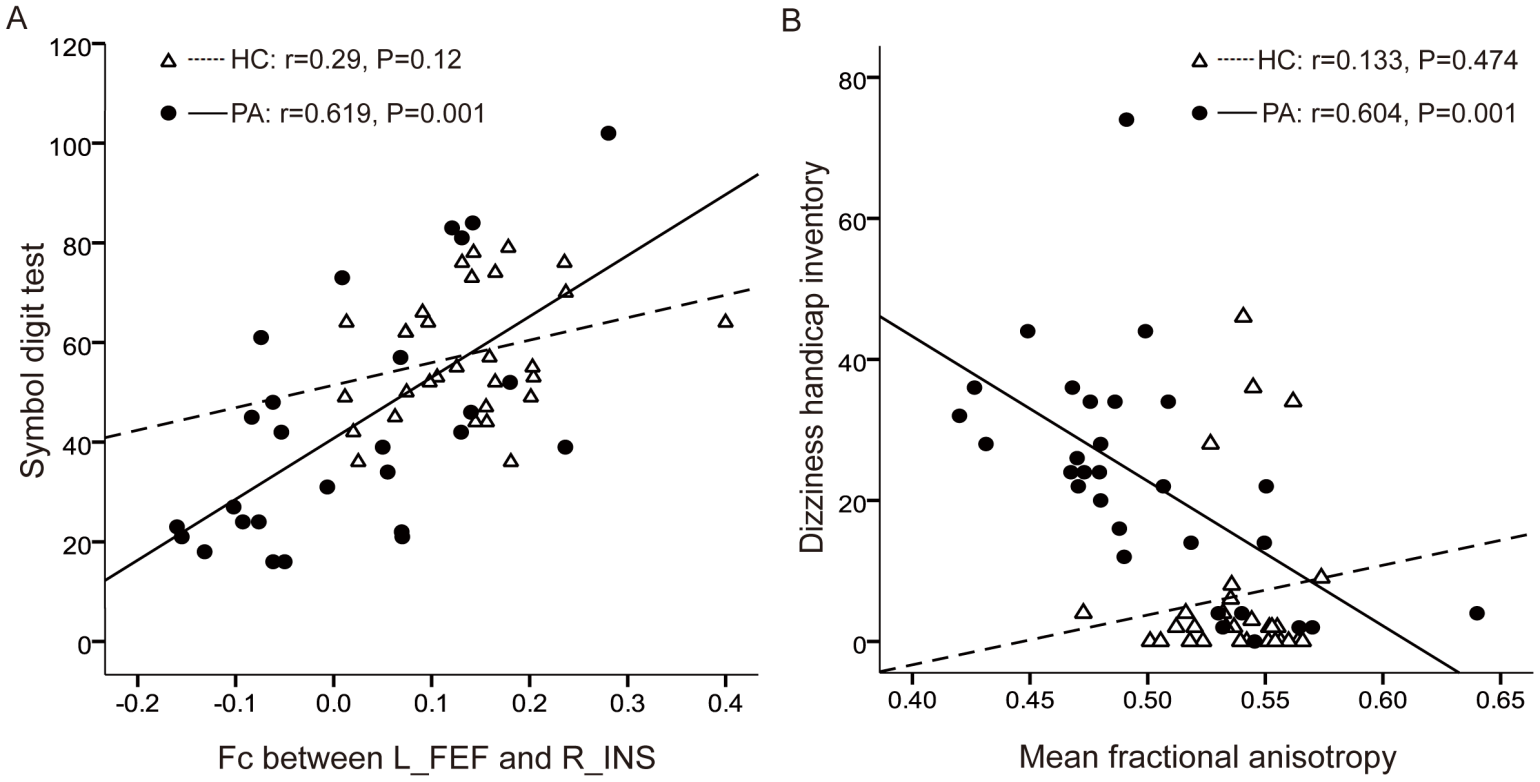
Simple linear regression relationships between connectivity strength and neurocognitive presentation. (A) The functional connectivity between the left frontal eye field (the seed in the dorsal attention network ipsilateral to carotid stenosis; the carotid stenotic side was flipped to the left in the patients) and the right insula (contralateral to carotid stenosis in the patients) is positively correlated with symbol digit test scores. (B) Mean fractional anisotropy is negatively correlated with dizziness handicap inventory scores. HC: healthy controls; PA: patients; L_FEF: left frontal eye field; R_INS: right insula.

The independent variables of multivariate regression analyses included the Fc values of the contralateral insula in the DAN, the contralateral MFG in the FPN, the contralateral S1 in the SMN and the ipsilateral MPF in the DMN, as well as age, sex, education levels, presence or absence of ICA stenosis (group) and mean FA. There was no co-linear interaction among the independent variables. Age (*P* = 0.001), the Fc strength at the right insula (*P* = 0.001) and education (*P* = 0.021) independently had linear relationships with attention performance (symbol digit test) (r^2^ = 0.668) after adjustments for group (*P* = 0.208) and sex (*P* = 0.244). Moreover, the mean FA value (*P* = 0.003; inversely linear relationship) and group (*P* = 0.008) were closely related to the severity of dizziness (DHI score) (r^2^ = 0.513). Figure 3 shows simple linear regression graphs between the Fc strength at the right insula and the attention performance, and the mean FA and the dizziness severity, respectively. There was no correlation between the mean FA and any regional significant Fc changes because of different locations. If a cut-off value of 1.5 SD below the mean of the healthy control group is adopted for the insula Fc (<0.05) or the mean FA (<0.53), 60% (18/30) or 70% (21/30) of the patients may be considered to have impaired connectivity, respectively. By contrast, 10% (3/30) or 27% (8/30) of the controls had impaired connectivity below these thresholds, respectively. Accordingly, a single connectivity measure is insufficient to distinguish individuals with MCI/VCIND from the healthy controls in terms of sensitivity or specificity. Nevertheless, the multivariate pattern classification (SVM) generated 92% predictive accuracy (AUC: 0.99) of individuals with MCI/VCIND (defined as having a delayed verbal recall score ≦8; feature weighting in the order of significance: age 30.7, education level −29.7, presence or absence of carotid stenosis 4.1, Fc in the DMN −1.3, Fc in the SMN −0.7, Fc in the DAN −0.5, Fc in the FPN −0.3 and mean FA −0.2) and 95% predictive accuracy (AUC: 0.997) of severe dizziness (defined as having a DHI score >24; feature weighting in the order of significance: education level −20.3, age 6.2, presence or absence of carotid stenosis 4.2, Fc in the SMN −1.7, Fc in the DMN −1.7, Fc in the FPN −0.8, Fc in the DAN −0.6 and mean FA −0.6). The minimum feature selection to accurately classify (>90%) mild cognitive impairment or severe dizziness can be achieved after removing mean FA, however, the original classifier gave the best classification/predictive accuracy. The most discriminative features in our classifiers are age, education level, presence or absence of carotid stenosis (group), and the Fc measures in the DMN and the SMN, depending on different neurocognitive outcomes. These results indicated a combinatorial contribution of multiple functional networks to cognitive performance and sense of equilibrium. Combining multiple demographic factors and functional image markers, we can identify individuals with MCI/VCIND for early treatment.

## Discussion

We characterized, for the first time to our knowledge, brain-wise connectivity and volumetric MRI measures in relation to neuropsychological performance in patients with severe, unilateral, asymptomatic carotid stenosis. These cognitively intact patients had significantly worse dizziness, poorer verbal (particularly episodic) memory and poorer executive and complex visuospatial performance than the controls. Approximately 40% of the patients had a delayed verbal recall score below our threshold and were considered MCI/VCIND. The brain-wise network comparisons showed distributed white matter disruption, in particular between the hemispheres, in the DAN (at the insula and the DLPFC contralateral to ICA stenosis), FPN (at the contralateral MFG, bilateral IPL and ipsilateral inferior frontal gyrus), SMN (at the contralateral S1 and contralateral SMC) and DMN (at the ipsilateral MPF), but not in the SN or VN. There were no significant differences in leukoaraiosis scores or brain volume between groups. Moreover, after controlling for confounding variables, the Fc strength at the insula (contralateral to ICA stenosis for the patients) showed a strong linear relationship with attention performance among all subjects, whereas the mean FA had a negative linear relationship with dizziness severity. Approximately 60%–70% of the patients were considered to have impaired connectivity below the threshold. The results may suggest that incipient disruption of interhemispheric long-range, non-hippocampal pathways precedes neurocognitive decline in patients with severe asymptomatic ICA stenosis.

VCIND may arise from heterogeneous causes, including silent infarcts, chronic hypoperfusion or both in combination[32], which can be treatable in contrast to neurodegenerative diseases. However, objective identification of brain dysfunction has been challenging at the early stages. DMN disruption has been commonly observed by rs-fMRI in patients with MCI[33], [34] and VCIND with subcortical vascular lesions[35]. Involvement of the executive attention network[34] has also been reported in MCI patients. Our patients unexpectedly showed diffuse disruption of multiple brain networks, which has not been reported previously. Among the six consistent functional networks we examined, the significantly affected regions in the DAN, FPN, SMN and DMN were mostly located in the ICA territory. In contrast, the relative preservation of the SN and VN, which are supplied by the anterior cerebral artery and the posterior cerebral artery, respectively, may be attributed to collaterals from the anterior communicating artery and the basilar artery, respectively. These findings suggest that connectivity impairment in severe ICA stenosis is likely region-specific and hypoperfusion-related.

The multivariate linear relationship between the Fc at the insula and the symbol digit test performance indicates that this Fc reflects insidious network insults related to early attention decline. The insula is a core region in the DAN that is involved in important processes of attention, pain, emotion and memory function[36], [37]. An enduring form of late-phase long-term potentiation has been noted in the brain slices of the adult mouse insular cortex following theta burst stimulation, supporting a role in insula-related memory[38]. The symbol digit test involves attention, execution, visual perception, memory and psychomotor speed[39]. Moreover, we found that the mean FA value was negatively related with dizziness severity and was significantly lower in the patients than in the controls, suggesting that mean FA may serve as a global image marker revealing diffuse network disruption in presymptomatic carotid stenosis. Dizziness is commonly but nonspecifically derived from various disorders of the peripheral vestibular system and central nervous system[40], as well as cardiogenic disorders or visual and psychiatric diseases[41]. Posturography has shown that MCI patients have poorer balance and higher sway speed than non-MCI controls after multivariate adjustments[42], suggesting the pivotal role of complex central integration pathways. We could not exclude comorbidity of other causes of dizziness and the relation to carotid stenosis needs further studies.

There are several limitations in this study. First, we did not evaluate brain perfusion in our subjects, so the causative role of hypoperfusion, at least partly, in cognitive decline requires further studies in asymptomatic carotid stenosis[14]. Second, we used 3,000 ms for TR during rs-MRI acquisition. A shorter TR of 2,500 ms, is currently also being used to improve the signal-to-noise ratio. Third, while mean FA represents the integrity of white matter tracts, Fc represents the temporal correlation of BOLD signals between ROI and gray matter regions. To obtain consistency between FA and Fc, fiber tractography may be needed to segment and quantify individual tract FA from the seed ROI of each network. Lastly, this is a cross-sectional case-control study. Longitudinal controlled studies of these patients with or without revascularization intervention may help to identify a causal relationship of ICA stenosis in MCI/VCIND.

## Conclusion

Patients with severe asymptomatic carotid stenosis may have extensive disruption in brain networks that correlates with neurocognitive decline even at a subclinical stage. Functional connectivity measures may facilitate the detection of network alterations and thus allow timely revascularization in patients with pre-symptomatic carotid stenosis.
